# FIRST-line support for Assistance in Breathing in Children (FIRST-ABC): a multicentre pilot randomised controlled trial of high-flow nasal cannula therapy versus continuous positive airway pressure in paediatric critical care

**DOI:** 10.1186/s13054-018-2080-3

**Published:** 2018-06-04

**Authors:** Padmanabhan Ramnarayan, Paula Lister, Troy Dominguez, Parviz Habibi, Naomi Edmonds, Ruth R. Canter, Jerome Wulff, David A. Harrison, Paul M. Mouncey, Mark J. Peters, on behalf of the United Kingdom Paediatric Intensive Care Society Study Group (PICS-SG)

**Affiliations:** 1 Children’s Acute Transport Service, Critical Care Division, Great Ormond Street Hospital NHS Foundation Trust, 26-27 Boswell Street, London, WC1N 3JZ UK; 2 Paediatric and Neonatal Intensive Care Unit, Critical Care Division, Great Ormond Street Hospital NHS Foundation Trust, London, UK; 3 Cardiac Intensive Care Unit, Critical Care Division, Great Ormond Street Hospital NHS Foundation Trust, London, UK; 4 Paediatric Intensive Care Unit, St Mary’s Hospital, Imperial College Healthcare NHS Trust, London, UK; 5 Paediatric Critical Care Unit, Royal London Hospital, Barts Health NHS Trust, London, UK; 6 Clinical Trials Unit, Intensive Care National Audit & Research Centre (ICNARC), Napier House, High Holborn, London, UK; 7 Respiratory, Critical Care and Anaesthesia Unit, Infection, Immunity and Inflammation Programme, UCL Great Ormond Street Institute of Child Health, London, UK

**Keywords:** High-flow nasal cannula therapy, Continuous positive airway pressure, Non-invasive respiratory support, Paediatric critical care

## Abstract

**Background:**

Although high-flow nasal cannula therapy (HFNC) has become a popular mode of non-invasive respiratory support (NRS) in critically ill children, there are no randomised controlled trials (RCTs) comparing it with continuous positive airway pressure (CPAP). We performed a pilot RCT to explore the feasibility, and inform the design and conduct, of a future large pragmatic RCT comparing HFNC and CPAP in paediatric critical care.

**Methods:**

In this multi-centre pilot RCT, eligible patients were recruited to either Group A (step-up NRS) or Group B (step-down NRS). Participants were randomised (1:1) using sealed opaque envelopes to either CPAP or HFNC as their first-line mode of NRS. Consent was sought after randomisation in emergency situations. The primary study outcomes were related to feasibility (number of eligible patients in each group, proportion of eligible patients randomised, consent rate, and measures of adherence to study algorithms). Data were collected on safety and a range of patient outcomes in order to inform the choice of a primary outcome measure for the future RCT.

**Results:**

Overall, 121/254 eligible patients (47.6%) were randomised (Group A 60%, Group B 44.2%) over a 10-month period (recruitment rate for Group A, 1 patient/site/month; Group B, 2.8 patients/site/month). In Group A, consent was obtained in 29/33 parents/guardians approached (87.9%), while in Group B 84/118 consented (71.2%). Intention-to-treat analysis included 113 patients (HFNC 59, CPAP 54). Most reported adverse events were mild/moderate (HFNC 8/59, CPAP 9/54). More patients switched treatment from HFNC to CPAP (Group A: 7/16, 44%; Group B: 9/43, 21%) than from CPAP to HFNC (Group A: 3/13, 23%; Group B: 5/41, 12%). Intubation occurred within 72 h in 15/59 (25.4%) of HFNC patients and 10/54 (18.5%) of CPAP patients (*p* = 0.38). HFNC patients experienced fewer ventilator-free days at day 28 (Group A: 19.6 vs. 23.5; Group B: 21.8 vs. 22.2).

**Conclusions:**

Our pilot trial confirms that, following minor changes to consent procedures and treatment algorithms, it is feasible to conduct a large national RCT of non-invasive respiratory support in the paediatric critical care setting in both step-up and step-down NRS patients.

**Trial registration:**

clinicaltrials.gov, NCT02612415. Registered on 23 November 2015.

**Electronic supplementary material:**

The online version of this article (10.1186/s13054-018-2080-3) contains supplementary material, which is available to authorized users.

## Background

Respiratory support is the most common organ support therapy provided in paediatric intensive care units (PICUs); nearly 75% of the 18,000 children admitted annually to PICUs in the United Kingdom and Ireland receive some form of respiratory support [1]. Over the past decade, concerns regarding the complications of invasive ventilation (IV) have prompted greater use of non-invasive respiratory support (NRS) modes such as continuous positive airway pressure (CPAP) [2–4]. Although the use of NRS has been shown to improve patient outcomes in randomised controlled trials (RCTs) in adult and neonatal intensive care [5–8], there is a dearth of RCTs in the PICU setting [9, 10].

CPAP has traditionally been used as the first-line NRS mode in two different clinical scenarios: 1) to prevent acutely ill children needing intubation and invasive ventilation (step-up NRS); and 2) to avoid re-intubation after extubation (step-down NRS) [11]. However, the use of CPAP in children is frequently limited by discomfort due to the need for a tight-fitting interface (face mask, hood, or nasal prongs) and the need for close monitoring to identify potential complications such as air leaks. More recently, an alternate mode of NRS, high-flow nasal cannula therapy (HFNC), has gained popularity since it is easy to use and well tolerated by patients [12–14]. Single-centre studies from the United States and Canada and audit data from the United Kingdom indicate that 16–35% of PICU admissions currently receive HFNC at some point during their stay [15–17]. Through diverse mechanisms such as reduction of airway resistance, reduction of dead space by nasopharyngeal washout with fresh gas and delivery of positive airway pressure (“CPAP effect”), HFNC has been shown to reduce the work of breathing and improve oxygenation and ventilation in children [18–23]. In single-centre observational studies, the use of HFNC has also been shown to be associated with a dramatic reduction in the need for intubation and invasive ventilation compared with historical controls [24–26]. However, there are few RCTs comparing HFNC with CPAP in the PICU setting [27–29].

The need for RCT evidence to support the clinical and cost effectiveness of HFNC was highlighted as a priority area in a recent European consensus statement of mechanical ventilation in children [30]. However, conducting such an RCT in a large group of critically ill children with diverse pathologies may be challenging, not least because equipoise among clinicians regarding the risks and benefits of HFNC may already be shifting [31]. To explore the feasibility of performing a future pragmatic RCT, and to inform its design and conduct, we conducted a multi-centre pilot RCT comparing CPAP and HFNC in critically ill children.

## Methods

A detailed description of the Methods has been published previously [32]. The study protocol is available at https://www.icnarc.org/Our-Research/Studies/First-Abc/Study-Documents.

### Study design and oversight

We conducted a pragmatic, open, multi-centre pilot RCT. The trial was sponsored by Great Ormond Street Hospital NHS Foundation Trust and co-ordinated by the Clinical Trials Unit (CTU) at the Intensive Care National Audit & Research Centre (ICNARC). The study received approval from the Health Research Authority (Integrated Research Application System, ref. 185074, and the National Research Ethics Service Committee North East – Tyne and Wear South, ref. 15/NE/0296). The trial was registered on clinicaltrials.gov (NCT02612415) prior to patient recruitment. As per Sponsor guidance, no formal data monitoring and ethics committee was established.

### Research sites and participants

Patients were recruited at three PICUs in London, UK, with a combined admission rate of 2500 annually and a baseline NRS usage rate of 15–35% [1]. Only one PICU had a written clinical guideline for the use of HFNC and CPAP.

Inclusion criteria were age > 36 weeks corrected for gestation and < 16 years, and the patient deemed by the treating clinician to require NRS either for an acute illness (Group A: step-up NRS) or after extubation (Group B: step-down NRS). Step-down NRS could be provided as a planned procedure immediately after extubation (‘planned’) or prompted by clinical deterioration within 72 h after extubation (‘rescue’). All patients in Group A and Group B ‘rescue’ were required to satisfy one or more objective clinical criteria for respiratory support: a) hypoxia (oxygen saturation < 92% in fraction of inspired oxygen > 0.40, or equivalent); b) acute respiratory acidosis (pH < 7.3 with a concomitant partial pressure of carbon dioxide (pCO_2_) > 6.5 kPa); and/or c) moderate respiratory distress (use of accessory muscles, subcostal and intercostal recession, tachypnoea for age, grunting). Exclusion criteria were: 1) deemed by the treating clinician to require immediate intubation/invasive ventilation due to severe hypoxia, acidosis and/or respiratory distress, upper airway obstruction or recurrent apnoeas; 2) tracheostomy in place; 3) pre-existing air-leak syndrome (pneumothorax, pneumomediastinum, subcutaneous emphysema); 4) mid-facial/craniofacial anomalies (unrepaired cleft palate, choanal atresia) or recent craniofacial surgery; 5) agreed limitation of intensive care treatment plan in place (‘not for intubation’); 6) domiciliary ventilation prior to PICU admission; 7) managed on NRS in the preceding 24 h; 8) previously recruited to the study during the same PICU admission; or 9) unable to be treated with NRS due to unavailability of device or appropriate interface.

### Randomisation and blinding

Patients were randomised as soon as study eligibility was confirmed. Pre-randomisation stratification was by group (A or B) and study site. Eligible patients were randomised to either CPAP or HFNC (1:1) using sequentially numbered, sealed, opaque envelopes available at each centre. The randomisation sequence was computer-generated by the trial statistician with variable block sizes to strengthen allocation concealment. The study was not blinded, since CPAP and HFNC are both already used in practice and recognisable by clinical staff.

### Intervention and control

Any commercially available Conformité Européene (CE)-marked medical device could be used to deliver HFNC and CPAP. Sites were allowed to use any CPAP interface (helmet, nasal prong, mask) as per their usual practice. The study protocol specified clinical criteria and procedures for the initiation, maintenance, and weaning of HFNC and CPAP (See Additional file 1: Figure S1 and Additional file 2: Figure S2). As per current practice, clinicians could consider stopping HFNC and switching to CPAP, or from CPAP to HFNC, if the patient met pre-specified criteria (crossover). Other treatments were given in accordance with standard practice at the sites; children who failed to improve on CPAP/HFNC could be managed with other modes of NRS (escalation) before intubation and ventilation as per the treating clinician’s discretion.

### Consent

We utilised a mixed consent model (prospective and deferred) appropriate to the nature of the clinical situation (planned or emergency initiation of NRS). For Group A, the site research team approached parents/guardians as soon as appropriate after randomisation (usually 24–48 h) to seek consent for continuation in the trial and use of study data. Deferred consent (or ‘research without prior consent’) is now a common consent methodology in emergency care trials and has been shown to be acceptable to parents/guardians as well as clinicians [33–35]. A postal ‘opt-out’ consent procedure was employed where participants were either discharged or died prior to consent being obtained. Prior to extubation (Group B), the site research team provided detailed written information to all parents/guardians of children receiving invasive ventilation on PICU. If NRS was started as a ‘planned’ treatment following extubation, written consent was obtained from parents/guardians before randomisation; if NRS was delivered as a rescue intervention after extubation, written consent was deferred.

### Outcomes

The outcome measures related to determining the feasibility of a future RCT. They were: 1) number of eligible patients in Groups A and B; 2) proportion of eligible patients randomised; 3) proportion of parents/guardians consenting to the study (prospective and deferred); 4) adherence to study protocol in terms of initiation, maintenance, and weaning of HFNC/CPAP; 5) use of a modified COMFORT score to assess patient tolerance; and 6) completion of a validated PSS:PICU questionnaire to measure parental stress 24 h after starting NRS [36]. Adverse events were documented. Data were collected on several patient outcomes to inform the choice of an appropriate primary outcome measure for the definitive trial, such as rate of crossover, rate of escalation, rate of treatment failure (crossover or escalation), rate of intubation, length of stay on PICU and in hospital, length of invasive and non-invasive respiratory support, and PICU and hospital mortality.

### Data collection

Study data, including serious adverse events, were collected and managed using Research Electronic Data Capture (REDCap) electronic data capture tools managed by the ICNARC Clinical Trials Unit (CTU) [37]. Sites collected data on patient demographics at randomisation and routine clinical observations at baseline and hourly for the first 6 h, then at 12, 24, 36, 48, and 72 h until the end of the assigned treatment (or crossover, escalation, or intubation/ventilation). A consent questionnaire was administered to all parents/guardians irrespective of whether they consented to the trial [33, 34, 38].

### Statistical analysis

No formal sample size calculations were performed for this pilot RCT; instead, sample size was determined to be adequate to estimate critical parameters to be tested to a necessary degree of precision [39]. Based on audit data, we expected to recruit around 120 study patients (Group A: 40; Group B: 80) over a 6-month period.

A statistical analysis plan was developed a priori (available at https://www.icnarc.org/Our-Research/Studies/First-Abc/Study-Documents). Statistical analyses were based on the intention-to-treat principle. All tests used were two-sided with significance level set at *p* < 0.05. Analyses were conducted using Stata/SE Version 14.0 (StataCorp, College Station, USA).

## Results

**Fig. 1:**
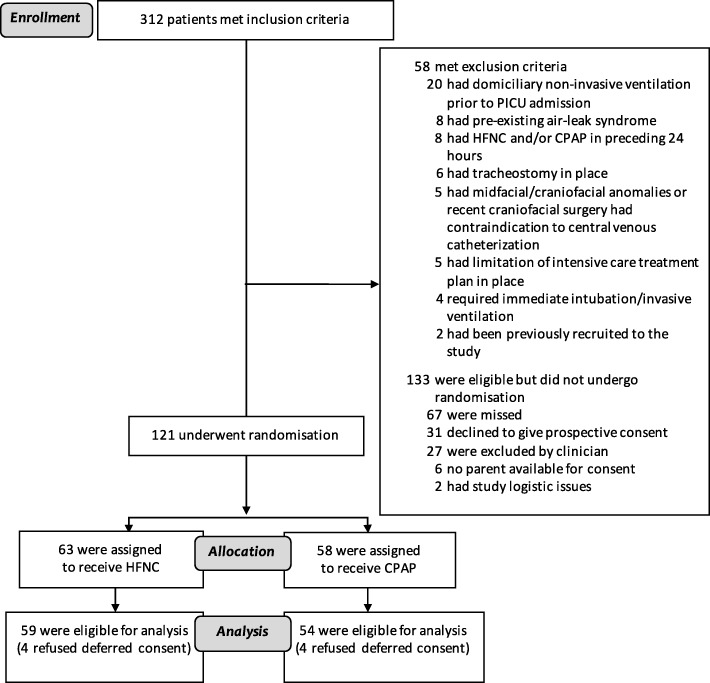
CONSORT flow diagram. CPAP continuous positive airway pressure, HFNC high-flow nasal cannula, PICU paediatric intensive care unit

The trial was conducted over 10 months (December 2015 to October 2016), although sites started recruitment in a staggered fashion due to delays in local approvals. Of the 312 patients who met the inclusion criteria, 58 were excluded, leaving 254 eligible participants (81.4%). Of these, 121 patients were randomised (HFNC: 63; CPAP: 58). Since consent to continue in the study was refused in eight cases, 113 patients were included in the intention-to-treat analysis (see Fig. 1 for CONSORT diagram).

### Number of eligible patients and proportion randomised

As shown in Table 1, there were more eligible patients in Group B than in Group A (199 versus 55). A higher proportion of Group A patients met exclusion criteria (24/79, 30.4%) compared with Group B (34/233, 14.6%). Nearly half of all eligible patients were randomised (121/254, 47.6%), although this rate differed by group (Group A: 60%; Group B: 44%). The overall recruitment rate was 1 patient per site per month for Group A and 2.8 patients per site per month for Group B. A large proportion of eligible participants not randomised were missed (66/133, 49.6%), usually due to inadequate research staff present during weekends and out-of-hours. Exclusion criteria and reasons for not randomising eligible patients are shown in Additional file 3: Table S1.

**Table 1:** Number of patients screened, eligible, randomised, and analysed by site

Variables	Site A	Site B	Site C	Total
Number screened, *n* (%)				
Group A	32 (20.0)	45 (73.8)	2 (2.2)	79 (25.3)
Group B				
Planned	74 (46.3)	6 (9.8)	87 (95.6)	167 (53.5)
Rescue	54 (33.8)	10 (16.4)	2 (2.2)	66 (21.1)
Total	160 (100)	61 (100)	91 (100)	312 (100)
Number of eligible patients, *n* (% of screened)				
Group A	27/32 (84.3)	27/45 (60.0)	1/2 (50)	55/79 (69.6)
Group B				
Planned	65/74 (87.8)	2/6 (33.3)	70/87 (80.5)	137/167 (82.0)
Rescue	52/54 (96.3)	9/10 (90.0)	1/2 (50)	62/66 (93.9)
Total	144/160 (88.1)	38/61 (62.3)	72/91 (79.1)	254/312 (81.4)
Number of patients randomised, *n* (% of eligible)				
Group A	12/27 (44.4)	21/27 (77.8)	0/1 (0)	33/55 (60.0)
Group B				
Planned	24/65 (36.9)	1/2 (50.0)	27/70 (38.6)	52/137 (38.0)
Rescue	26/52 (50.0)	9/9 (100)	1/1 (100)	36/62 (58.1)
Total	62/144 (43.1)	31/38 (81.6)	28/72 (38.9)	121/254 (47.6)
Number of patients analysed, *n* (% of randomised)				
Group A	11/12 (91.7)	18/21 (85.7)	0/0 (0)	29/33 (87.9)
Group B				
Planned	24/24 (100)	1/2 (50.0)	27/27 (100)	52/52 (100)
Rescued	23/26 (88.5)	8/9 (88.9)	1/1 (100)	32/36 (88.9)
Total	58/62 (93.5)	27/31 (87.1)	28/28 (100)	113/121 (93.4)

### Consent rate

Overall, 113 out of 151 parents approached consented to participate in the study (74.8%), with differences between Groups A and B (87.9% and 71.2%, respectively). As shown in Additional file 3: Table S2, the rate of consent varied by consent model (prospective consent in Group B ‘planned’: 63.4%; deferred consent in Group A and Group B ‘rescue’: 88.4%). There was no significant difference in the rate of refusal of deferred consent based on the randomised treatment (HFNC: 93.7%; CPAP: 93.1%). Consent questionnaires returned (*n* = 20) indicated that deferred consent was acceptable to most parents (14/17, 82%). One parent commented that “research should not delay extubation” (Group B ‘planned’) indicating that attempting to obtain consent prior to extubation may in fact delay clinical management (Additional file 3: Table S3).

### Baseline characteristics

Baseline characteristics of patients recruited to Groups A and B are shown in Table 2. There was some imbalance in age and consequently in weight. Nearly half of patients in Group A had parenchymal lung disease as their primary diagnosis while this proportion was lower in Group B. Modified COMFORT scores (where available) were similar.

**Table 2:** Baseline characteristics and clinical variables of patients recruited to Groups A and B by treatment group

Variables	Group A		Group B	
	HFNC	CPAP	HFNC	CPAP
	*n* = 16(55.2%)	*n* = 13(44.8%)	*n* = 43(51.2%)	*n* = 41(48.8%)
*Demography*				
Age (years)	*n* = 16	*n* = 13	*n* = 43	*n* = 40
Median (IQR)	1.5 (0.6–7.0)	0.8 (0.7–1.0)	0.7 (0.2–3.0)	0.5 (0.1–1.0)
Age group, *n* (%)				
<1 year	5 (31.3)	7 (53.8)	24 (55.8)	28 (70.0)
1 to 2 years	5 (31.3)	5 (38.5)	8 (18.6)	7 (17.5)
3 to 4 years	1 (6.3)	0 (0.0)	2 (4.7)	0 (0.0)
5 to 9 years	2 (12.5)	0 (0.0)	5 (11.6)	3 (7.5)
10 years and over	3 (18.8)	1 (7.7)	4 (9.3)	2 (5.0)
Gender, *n* (%)				
Female	8 (50.0)	7 (58.3)	27 (62.8)	14 (35.0)
Male	8 (50.0)	5 (41.7)	16 (37.2)	26 (65.0)
Weight (kg)	*n* = 16	*n* = 13	*n* = 43	*n* = 41
Median (IQR)	11.4 (6.1–17.1)	9.0 (7.0–12.6)	7.5 (5.0–16.3)	5.8 (3.6–10.0)
*Diagnosis*				
Primary reason for PICU admission, *n* (%)				
Apnoea	1 (6.3)	0 (0.0)	1 (2.3)	0 (0.0)
Asthma/wheeze	3 (18.8)	0 (0.0)	1 (2.3)	1 (2.4)
Bronchiolitis	2 (12.5)	4 (30.8)	8 (18.6)	11 (26.8)
Cardiac	0 (0.0)	0 (0.0)	5 (11.6)	3 (7.3)
Lung disease	7 (43.8)	7 (53.8)	13 (30.2)	9 (22.0)
Neurological	0 (0.0)	0 (0.0)	4 (9.3)	8 (19.5)
Neuromuscular disorder	0 (0.0)	0 (0.0)	2 (4.7)	0 (0.0)
Sepsis	2 (12.5)	1 (7.7)	4 (9.3)	0 (0.0)
Upper airway obstruction	1 (6.3)	1 (7.7)	4 (9.3)	4 (9.8)
Other	0 (0.0)	0 (0.0)	1 (2.3)	5 (12.2)
Length of invasive ventilation prior to extubation (days)			*n* = 41	*n* = 40
Median (IQR)			3.0 (2.0–5.0)	4.0 (2.0–6.0)
Modified COMFORT score at baseline	*n* = 7	*n* = 4	*n* = 15	*n* = 10
Median (IQR)	17.0 (12.0–22.0)	15.0 (15.0–17.5)	17.0 (14.0–19.0)	17.0 (14.0–22.0)
*Physiology at baseline*				
Respiratory rate (breaths/min)	*n* = 14	*n* = 12	*n* = 38	*n* = 36
Median (IQR)	40.5 (33.0–55.0)	42.0 (39.0–57.0)	31.5 (27.0–40.0)	33.0 (24.5–40.0)
Heart rate (beats/min)	*n* = 15	*n* = 12	n = 38	*n* = 36
Median (IQR)	148.0 (128.0–156.0)	147.5 (125.0–166.0)	138.5 (112.0–152.0)	146.5 (115.5–168.0)
SpO_2_ (%)	*n* = 14	*n* = 12	*n* = 37	*n* = 35
Median (IQR)	97.0 (94.0–98.0)	99.0 (96.5–100.0)	98.0 (95.0–99.0)	98.0 (96.0–100.0)
PaO_2_ (kPa)	*n* = 0	*n* = 0	*n* = 8	*n* = 5
Median (IQR)	–	–	8.8 (5.4,11.4)	9.5 (6.8,10.4)
FiO_2_	*n* = 14	*n* = 12	*n* = 37	*n* = 33
Median (IQR)	0.7 (0.4–0.8)	0.5 (0.4–0.7)	0.3 (0.3–0.5)	0.3 (0.3–0.4)
pH	*n* = 6	*n* = 3	*n* = 14	*n* = 11
Median (IQR)	7.4 (7.4–7.4)	7.3 (7.3–7.4)	7.4 (7.4–7.5)	7.3 (7.3–7.4)
pCO_2_ (kPa)	*n* = 6	*n* = 3	*n* = 14	*n* = 11
Median (IQR)	5.9 (5.4–6.6)	5.5 (4.8–7.2)	5.2 (4.6–6.8)	7.1 (6.1–7.7)
*Work of breathing*				
Respiratory distress, *n* (%)				
None	0 (0.0)	0 (0.0)	16 (53.3)	13 (44.8)
Mild	3 (21.4)	1 (12.5)	6 (20.0)	4 (13.8)
Moderate	8 (57.1)	4 (50.0)	3 (10.0)	9 (31.0)
Severe	3 (21.4)	3 (37.5)	5 (16.7)	3 (10.3)

CPAP continuous positive airway pressure, FiO_2_ fraction of inspired oxygen, HFNC high-flow nasal cannula, IQR interquartile range, PaO_2_ partial pressure of oxygen, pCO_2_ partial pressure of carbon dioxide, PICU paediatric intensive care unit, SD standard deviation, SpO_2_ peripheral capillary oxygen saturation

### Adherence to protocol

**Fig. 2:**
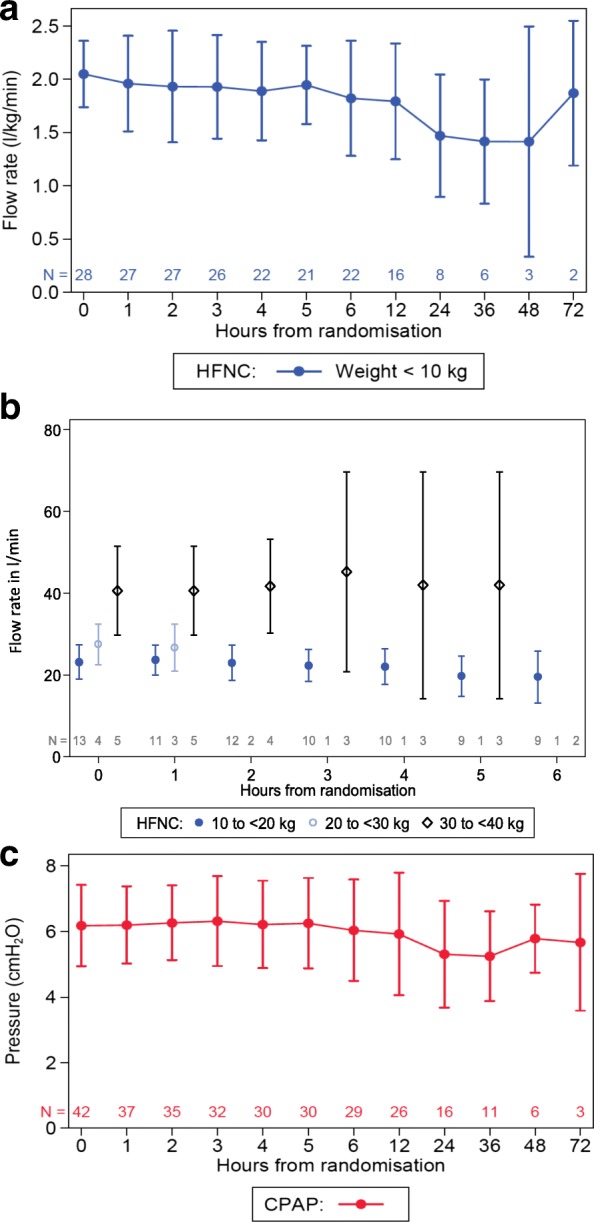
High-flow nasal cannula (HFNC) gas flow rates and continuous positive airway pressure (CPAP) measurements for study participants. **a** HFNC gas flow rate for patients weighing < 10 kg; **b** HFNC gas flow rate for patients weighing > 10 kg; **c** CPAP pressure for all patients

The majority of patients randomised to the study started the allocated treatment (HFNC: 55/59, 93.2%; CPAP: 48/54, 88.9%). The main reason for not starting the allocated treatment in Group A (2/10 patients) was clinical deterioration necessitating emergency intubation, while the reason in Group B ‘planned’ (7/10 patients) was a clinical decision at the time of extubation that the child did not actually require NRS. The median time between randomisation and starting emergency HFNC or CPAP was less than 20 min; for Group B ‘planned’, HFNC was started within a median of 1 h (interquartile range (IQR) 0.2–4.5) and CPAP in 2 h (IQR 0.4–11.7). Recommended gas flow rates were established by hour 2 in the majority of HFNC patients (39/52, 75%); similarly, adherence to the specified pressure for CPAP by hour 2 was good (29/43, 67.4%). Mean HFNC rate and CPAP pressures during the study period are shown in Fig. 2.

### Patient comfort and parental stress

Mean modified COMFORT scores in the first 6 h, although available at less than half of all eligible time points, were higher for patients who were clinically assessed to be not tolerating CPAP compared with those who were not tolerating HFNC (19.0, standard deviation (SD) 4.4 versus 15.3, SD 3.1) as shown in Additional file 3: Table S4. PSS:PICU scores, available in just a third of patients in both treatment groups, were higher in CPAP patients (median 2.1, IQR 1.8–2.5) compared with HFNC patients (median 1.8, IQR 1.5–2.2).

### Safety

Overall, both treatments were safe with no complications of air-leak syndrome reported (Additional file 3: Table S5). None of the three cases of respiratory/cardiac arrest recorded in the CPAP group were judged to be related to the treatment itself.

### Potential outcome measures

As shown in Additional file 4: Figure S3, a third of patients randomised to HFNC were either switched to CPAP (16/59, 27.1%) or directly escalated to other forms of ventilation within 72 h of randomisation (6/59, 10.2%), whereas this occurred less frequently in CPAP patients (switched to HFNC: 8/54, 14.8%; directly escalated: 8/54, 14.8%). Reasons are shown in Additional file 3: Table S6. A greater proportion of HFNC patients needed intubation within 72 h of randomisation compared with CPAP patients (15/59, 25.4%, versus 10/54, 18.5%; risk difference 6.9%, 95% confidence interval (CI) –8.3 to 22.1). PICU mortality was 5.1% in HFNC patients compared with 3.7% in CPAP patients. A summary of the effect estimates of key patient outcomes is shown in Table 3.

**Table 3:** Comparison of outcomes by treatment group for Group A and Group B

Outcome	Group A			Group B			*P* value^a^
	HFNC	CPAP	Effect estimates (95% CI)	HFNC	CPAP	Effect estimates (95% CI)	
	*n* = 16	*n* = 13		*n* = 43	*n* = 41		
*Intubation within 72 h*							
*n* (%)	6/16 (37.5)	2/13 (15.4)	Risk ratio:	9/43 (20.9)	8/41 (19.5)	Risk ratio:	0.331
			2.44 (0.59–10.12)			1.07 (0.46–2.51)	
			Risk difference (%):			Risk difference (%):	
			22.1 (−8.7 to 52.9)			1.4 (−15.8 to 18.6)	
*Crossover or escalation within 72 h*							
n (%)	8/16 (50.0)	4/13 (30.8)	Risk ratio:	14/43 (32.6)	12/41 (29.3)	Risk ratio:	0.517
			1.63 (0.63–4.21)			1.11 (0.59–2.11)	
			Risk difference (%):			Risk difference (%):	
			19.2 (−15.8 to 54.3)			3.3 (−16.5 to 23.0)	
*Length of PICU stay from randomisation (days):*							
Mean (SD)	6.7 (5.8)	5.9 (5.2)	Mean difference:	8.6 (14.9)	6.1 (7.0)	Mean difference:	0.696
			0.7 (−3.5 to 5.0)			2.5 (−2.6 to 7.6)	
Median (IQR)	5.4 (2.0–10.4)	4.4 (2.3–6.3)		3.2 (1.2–6.8)	4.1 (2.1–5.9)		
*Length of hospital stay (days)*							
Mean (SD)	24.1 (26.1)	31.9 (29.1)	Mean difference:	24.0 (37.9)	21.4 (49.2)	Mean difference:	0.556
			−7.8 (−28.9 to 13.3)			2.6 (−17.2 to 22.4)	
Median (IQR)	15 (5–37)	20 (9–41)		7 (4–19)	7 (5–15)		
*Length of invasive ventilation from first escalation (days)*							
Mean (SD)	1.5 (3.3)	1.8 (5.2)	Mean difference:	1.5 (4.8)	1.4 (4.8)	Mean difference:	0.865
			−0.3 (−3.6 to 3.0)			0.1 (−2.0 to 2.1)	
Median (IQR)	0.0 (0.0–1.1)	0.0 (0.0–0.0)		0.0 (0.0–0.0)	0.0 (0.0–0.0)		
*Length of randomised treatment (days)*							
Mean (SD)	0.8 (0.8)	1.1 (1.2)	Mean difference:	0.9 (1.3)	0.9 (1.7)	Mean difference:	0.651
			−0.3 (−1.1 to 0.5)			−0.0 (− 0.7 to 0.6)	
Median (IQR)	0.5 (0.1–1.4)	0.8 (0.3–1.6)		0.6 (0.1–0.8)	0.4 (0.0–1.0)		
*Ventilator-free days at day 28*							
Mean (SD)	19.6 (9.0)	23.5 (7.3)	Mean difference:	21.8 (8.0)	22.2 (8.5)	Mean difference:	0.317
			−4.0 (−10.3 to 2.4)			−0.4 (−4.0 to 3.2)	
*PICU mortality*							
n (%)	2/16 (12.5)	1/13 (7.7)	Risk ratio:	1/43 (2.3)	1/41 (2.4)	Risk ratio:	0.770
			1.63 (0.17–15.99)			0.95 (0.06–14.75)	
			Risk difference (%):			Risk difference (%):	
			4.8 (−16.9 to 26.5)			−0.1 (−6.6 to 6.4)	
*Hospital mortality*							
n (%)	2/16 (12.5)	1/13 (7.7)	Risk ratio:	3/42 (7.1)	1/40 (2.5)	Risk ratio:	0.728
			1.63 (0.17–15.99)			2.86 (0.31–26.34)	
			Risk difference (%):			Risk difference (%):	
			4.8 (−16.9 to 26.5)			4.6 (−4.5 to 13.8)	

CI confidence interval, CPAP continuous positive airway pressure, HFNC high-flow nasal cannula, IQR interquartile range, PICU paediatric intensive care unit, SD standard deviation

^a^Interaction between Group A vs Group B and effect of treatment

## Discussion

In this multi-centre pilot RCT, we successfully enrolled nearly half of all eligible critically ill children with an average recruitment rate of 3.8 participants per site per month. Consent was obtained in 75% of participants, in line with previous paediatric emergency trials. There was good clinician adherence to the HFNC and CPAP study algorithms. Both treatments were safe and, although not powered to test for significance, outcome data suggested that the rate of intubation and length of respiratory support were potentially important outcomes to consider in a future RCT.

In this pilot trial, performed in advance of a large definitive RCT, we clarified three important areas of uncertainty: whether PICU clinicians would be willing to randomise participants considering that HFNC may be superseding CPAP as the first-line choice for NRS in paediatric settings [31]; whether the study algorithms were acceptable to clinicians and practical to use, considering the variability in current practice relating to the use of HFNC/CPAP [40]; and whether we could identify a suitable patient-centred and clinically relevant primary outcome measure, considering that previous RCTs of HFNC have focussed on surrogate outcome measures such as crossover or treatment failure [29, 41–43]. We found that the main reason for not randomising eligible patients was because they were missed, rather than clinician preference; clinicians started the allocated treatment in nearly all patients (HFNC 93% and CPAP 89%) and followed the recommended gas flow rate/pressure in the majority of cases. Furthermore, and irrespective of whether patients were switched or escalated to other treatments, the choice of first-line NRS mode influenced the rate of intubation and overall length of respiratory support, indicating that they might be candidate outcome measures for a future RCT.

The design and conduct of a future RCT will be influenced by this study in several ways. Since the two main clinical scenarios in which NRS is used in critically ill children (step-up and step-down) are different in terms of patient case mix and distribution of potential outcomes, and results from one may not be easily generalisable to the other, a future RCT should consider studying both populations, powered separately, within a common trial infrastructure to maximise efficiency of design and conduct. Since there were more eligible children in Group B than in Group A from PICUs involved in this study, and anticipated recruitment to Group A might be slower than Group B, the participation of a mix of paediatric critical care units (where step-up care is provided more frequently) as well as intensive care units is an important consideration. A future trial should also consider simplifying the consent process in light of parental questionnaire responses and since the requirement for clinicians to predict in advance whether they planned to start the patient on NRS post-extubation was prone to error: 7/10 cases where the allocated treatment was not started were in Group B ‘planned’. One solution might be to provide information sheets to parents/guardians of children in Group B before extubation but randomise participants only when eligibility (the clinical decision to start NRS) is confirmed after extubation, and to defer written consent. A definitive trial would also benefit from more explicit guidance on weaning HFNC, changes to the recommended HFNC flow rate to address practical issues related to nasal cannula size, and greater clinical discretion to set CPAP pressures. The low completion rate of modified COMFORT scores indicates that, if it were to be used in a future RCT, it needs to be incorporated into routine practice in participating PICUs to maximise data completion.

There are several strengths of this pilot RCT. First, it is the only report of a randomised comparison between HFNC and CPAP for step-up and step-down NRS in a group of critically ill children with diverse conditions. Second, the trial successfully addressed the main areas of uncertainty involved in conducting a large, efficient, pragmatic RCT of first-line mode of NRS in the paediatric critical care setting, providing valuable insights into the design of future RCTs in this area. As a pragmatic trial, we aimed to ensure that any research findings can be generalised to clinical practice across a wide spectrum of units. Third, clinical engagement with the trial was good and contributed to the high degree of adherence to study procedures seen. The main limitations relate to the pilot study design which precludes any firm conclusions regarding the effectiveness of HFNC or CPAP to be drawn from the data and the limited generalisability of the findings considering only three PICUs participated in the study.

## Conclusions

This multi-centre pilot RCT confirms that it is feasible to conduct a large pragmatic national clinical trial of non-invasive respiratory support in the PICU setting in both step-up and step-down NRS. Considerations for a future RCT include how to incorporate both populations, adoption of a uniform consent model that is practical and acceptable to clinicians and participants, and the choice of a suitable patient-centred and clinically relevant primary outcome measure.

## Additional files

Additional file 1: **Figure S1.** Study algorithm for the management of patients randomised to high-flow nasal cannula therapy. (PDF 207 kb)

Additional file 2: **Figure S2.** Study algorithm for the management of patients randomised to continuous positive airway pressure. (PDF 156 kb)

Additional file 3: **Table S1.** Exclusion criteria and reasons for not randomising eligible patients by group. **Table S2.** Number of patients approached for consent and consented by site. **Table S3.** Parents’ survey responses regarding the pilot trial consent process (*n* = 20). **Table S4.** Modified COMFORT score and use of sedative agents by treatment group. **Table S5.** Adverse events by treatment group. **Table S6.** Reasons and timing for crossover and escalation to intubation and invasive ventilation within 72 h by treatment group. (DOCX 42 kb)

Additional file 4: **Figure S3.** Crossover and escalation to invasive ventilation within 72 h of starting the randomised treatment by treatment group. (PDF 50 kb)
