# Supplementary figures and images for: FIRST-line support for Assistance in Breathing in Children (FIRST-ABC): a multicentre pilot randomised controlled trial of high-flow nasal cannula therapy versus continuous positive airway pressure in paediatric critical care
Source: Crit Care. 2018 Jun 4;22:144. doi: 10.1186/s13054-018-2080-3 (PMC5987627; doi:10.1186/s13054-018-2080-3)

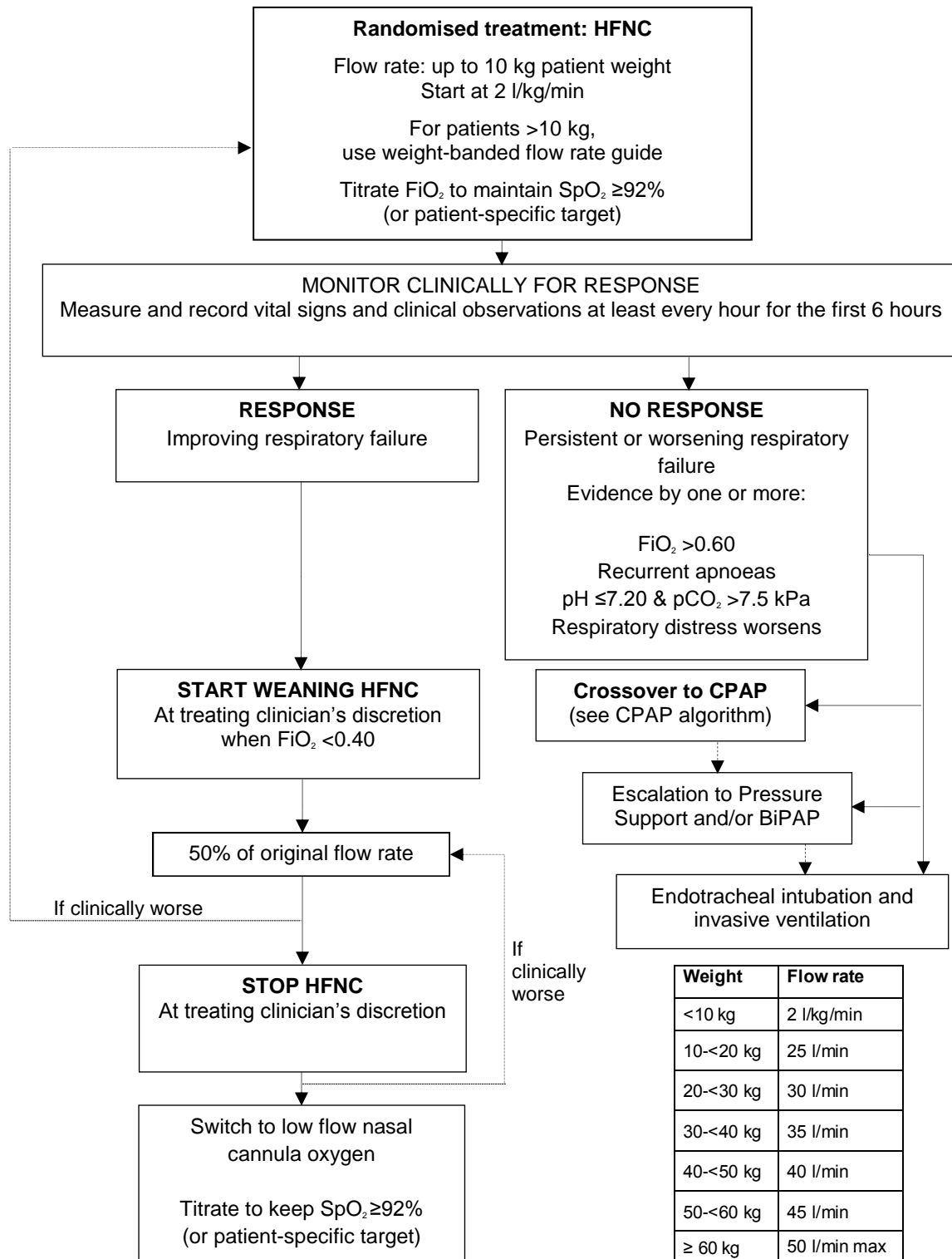

Supplement: Supplementary file 1 — Figure S1. Study algorithm for the management of patients randomised to high-flow nasal cannula therapy. (PDF 207 kb) [file 13054_2018_2080_MOESM1_ESM.pdf]

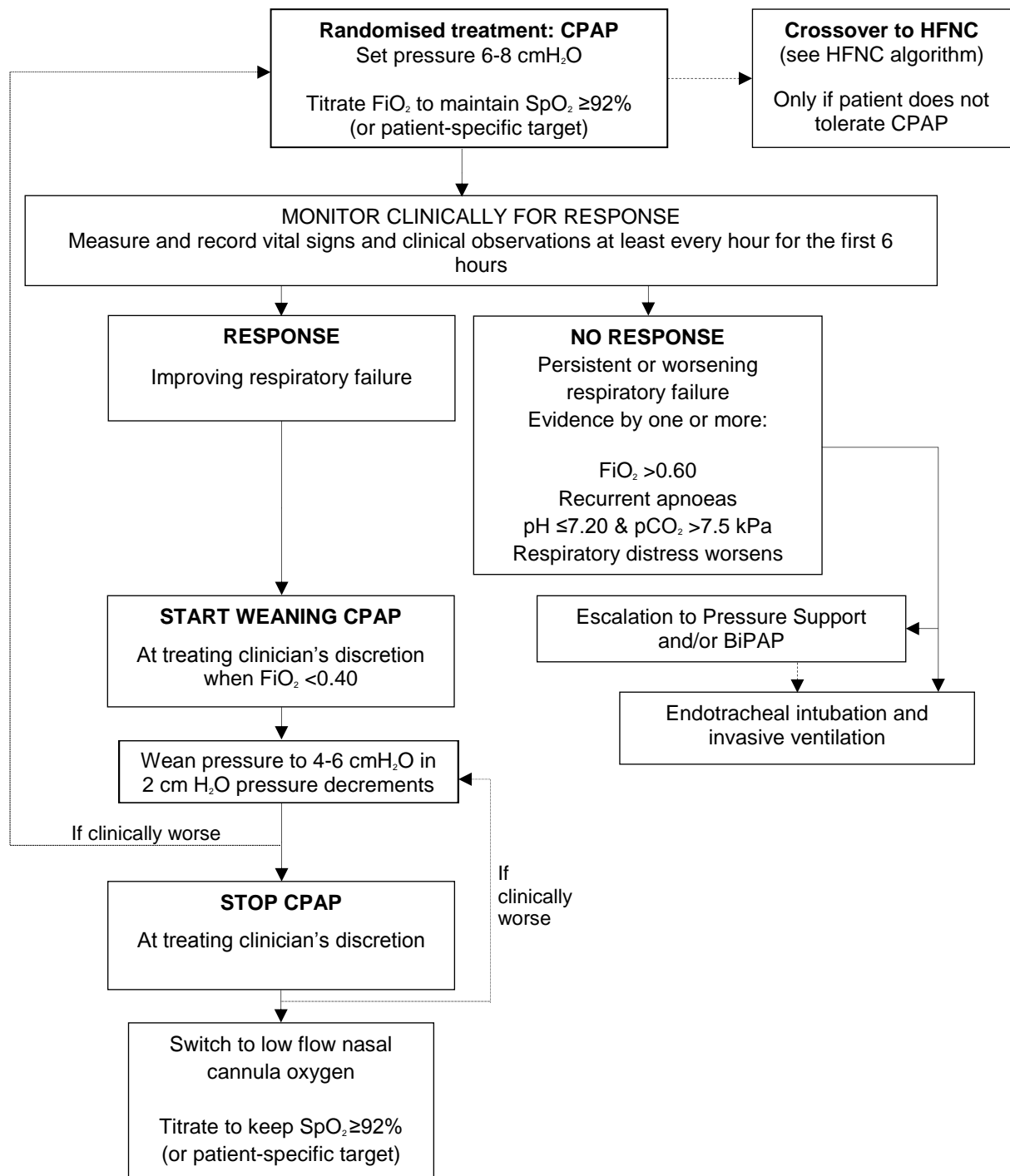

Supplement: Supplementary file 2 — Figure S2. Study algorithm for the management of patients randomised to continuous positive airway pressure. (PDF 156 kb) [file 13054_2018_2080_MOESM2_ESM.pdf]

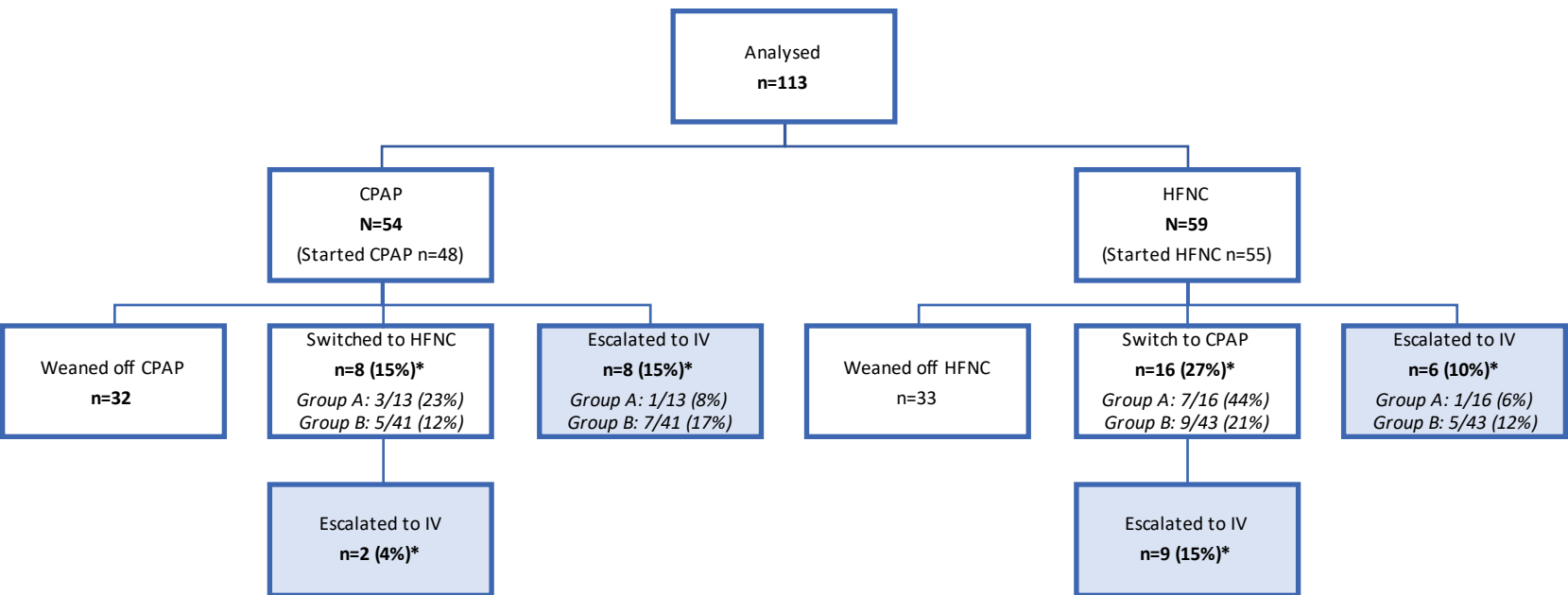

\* Percentage shown indicates proportion of children randomised to CPAP or HFNC (n/N)

Supplement: Supplementary file 4 — Figure S3. Crossover and escalation to invasive ventilation within 72 h of starting the randomised treatment by treatment group. (PDF 50 kb) [file 13054_2018_2080_MOESM4_ESM.pdf]
