# Supplementary material for: FIRST-line support for Assistance in Breathing in Children (FIRST-ABC): a multicentre pilot randomised controlled trial of high-flow nasal cannula therapy versus continuous positive airway pressure in paediatric critical care
Source: Crit Care. 2018 Jun 4;22:144. doi: 10.1186/s13054-018-2080-3 (PMC5987627; doi:10.1186/s13054-018-2080-3)
Supplement: Supplementary file 3 — Table S1. Exclusion criteria and reasons for not randomising eligible patients by group. Table S2. Number of patients approached for consent and consented by site. Table S3. Parents’ survey responses regarding the pilot trial consent process (n = 20). Table S4. Modified COMFORT score and use of sedative agents by treatment group. Table S5. Adverse events by treatment group. Table S6. Reasons and timing for crossover and escalation to intubation and invasive ventilation within 72 h by treatment group. (DOCX 42 kb) [file 13054_2018_2080_MOESM3_ESM.docx]

Table S1: Exclusion criteria and reasons for not randomising eligible patients by group

| **Reason** | **Group A** | **Group B**  **Planned** | **Group B**  **Rescue** | **Total** |
| --- | --- | --- | --- | --- |
| **Number of patients meeting exclusion criteria, n (% of screened)** |  |  |  |  |
| Required immediate intubation | 3/79 (3.8) | 1/167 (0.6) | 0 | 4/312 (1.3) |
| Tracheostomy in place | 0 | 6/167 (3.6) | 0 | 6/312 (1.9) |
| Pre-existing air-leak | 4/79 (5.1) | 4/167 (2.4) | 0 | 8/312 (2.6) |
| Facial anomalies/surgery | 1/79 (1.3) | 4/167 (2.4) | 0 | 5/312 (1.6) |
| Agreed limitation of intensive care | 1/79 (1.3) | 3/167 (1.8) | 1/66 (1.5) | 5/312 (1.6) |
| Domiciliary NRS prior to admission | 8/79 (10.1) | 10/167 (6.0) | 2/66 (3.0) | 20/312 (6.4) |
| Managed on HFNC/CPAP in previous 24 hours | 7/79 (8.9) | 0 | 1/66 (1.5) | 8/312 (2.6) |
| Previously recruited to the study | 0 | 2/167 (1.2) | 0 | 2/312 (0.6) |
| Unable to be treated with HFNC | 0 | 0 | 0 | 0 |
| Unable to be treated with CPAP | 0 | 0 | 0 | 0 |
| Total | 24/79 (30.4) | 30/167 (18.0) | 4/66 (6.0) | 58/312 (18.6) |
|  |  |  |  |  |
| **Number of eligible patients not randomised, n (% of eligible)** |  |  |  |  |
| Missed by research team | 14/55 (25.5) | 27/137 (19.7) | 25/62 (40.3) | 66/254 (26.0) |
| Excluded by clinician | 5/55 (1.0) | 22/137 (16.1) | 1/62 (1.6) | 28/254 (11.0) |
| Logistical issues | 3/55 (0.5) | 1/137 (0.7) | 0 | 4/254 (1.6) |
| Declined consent | NA | 30/137 (21.9) | NA | 30/254 (11.8) |
| Parent/guardian unavailable | NA | 5/137 (3.6) | NA | 5/254 (1.9) |
| Total | 22/55 (40.0) | 85/137 (62.0) | 26/62 (41.9) | 133/254 (52.4) |

Table S2: Number of patients approached for consent and consented by site

| **Variables** | **Site A** | **Site B** | **Site C** | **Total** |
| --- | --- | --- | --- | --- |
| **Number approached for consent, N (%)** |  |  |  |  |
| Group A | 12 | 21 | 0 | 33 |
| Group B |  |  |  |  |
| Planned | 29 | 1 | 52 | 82 |
| Rescue | 26 | 9 | 1 | 36 |
| Total | 67 | 31 | 53 | 151 |
| **Consent obtained, n (% of approached)** | | |  |  |
| Group A (deferred) | 11/12 (91.7) | 18/21 (85.7) | 0 | 29/33 (87.9) |
| Group B |  |  |  |  |
| Planned (prospective) | 24/29 (82.7) | 1/1 (100) | 27/52 (51.9) | 52/82 (63.4) |
| Rescue (deferred) | 23/26 (88.5) | 8/9 (88.9) | 1/1 (100) | 32/36 (88.9) |
| Total | 58/67 (86.6) | 27/31 (87.1) | 28/53 (52.8) | 113/151 (74.8) |

Table S3: Parents’ survey responses regarding the pilot trial consent process (N=20)

| **Statement** | **Agree**  **n (%)** | | **Neither agree nor disagree  n (%)** | | | **Disagree**  **n (%)** | |
| --- | --- | --- | --- | --- | --- | --- | --- |
| a. The doctor or nurse checked that it was a convenient time to discuss research before discussing FIRST-ABC | 18 | 95% | 1 | 5% | | 0 | 0% |
| b. I was initially surprised to find out that my child had already been entered into FIRST-ABC | 2 | 12% | 10 | 59% | | 5 | 29% |
| c. The information I received about FIRST-ABC was clear and straightforward to understand | 20 | 100% | 0 | 0% | | 0 | 0% |
| d. I understood why consent for my child’s participation in FIRST-ABC was sought after the treatment had been given | 16 | 84% | 2 | 11% | | 1 | 5% |
| e. I had enough opportunity to ask questions about FIRST-ABC | 20 | 100% | 0 | 0% | | 0 | 0% |
| f. I was satisfied with the deferred consent process for FIRST-ABC | 14 | 82% | 2 | 12% | | 1 | 6% |
| g. It was difficult to take in the information I was given about FIRST-ABC | 1 | 5% | 3 | 15% | | 16 | 80% |
| h. It was difficult to make a decision about FIRST-ABC | 3 | 15% | 2 | 10% | | 15 | 75% |
| i. I made this decision | 18 | 90% | 0 | 0% | | 2 | 10% |
| j. Someone took this decision away from me | 1 | 5% | 0 | 0% | | 19 | 95% |
| k. I was not in control of this decision | 1 | 5% | 0 | 0% | | 19 | 95% |
| l. The decision about the research was inappropriately influenced by others | 0 | 0% | 1 | 5% | | 19 | 95% |
| **Consent** | n | % |  |  | |  |  |
| Did you consent for your child to participate in FIRST-ABC? | | | | | | | |
| Yes | 19 | | | 95% | | | |
| No | 1 | | | 5% | | | |
| Reason for non-consent: My child needs regular suctioning and has previously found CPAP to be very uncomfortable. | | | | | | | |
| **Reason for consent** | **Identified as a reason** | | | | | | |
|  | **n=19** | | | | **%** | | |
| 1. To help my child | 13 | | | | 68% | | |
| 2. To help other children in the future | 19 | | | | 100% | | |
| 3. I felt that medical studies like FIRST-ABC are important | 18 | | | | 95% | | |
| 4. Because I trusted the doctor or nurse who explained FIRST-ABC | 13 | | | | 68% | | |
| 5.The treatment had already been given to my child | 9 | | | | 47% | | |
| 6. My child recovered | 6 | | | | 32% | | |
| 7. I didn't feel comfortable saying no to the nurse or doctor who explained | 3 | | | | 16% | | |
| 8. Other | 2 | | | | 11% | | |
| Other reason: I would have wanted my child to have the treatment that she was randomly allocated. I may not have been as happy if she was allocated opti-flow. (1 other reason given) | | | | | | | |
|  | | | | | | | |
| **Other comments** | | | | | | | |
| The initial information should include pictures of both devices. | | | | | | | |
| Research should not delay extubation. Maybe doctors could talk through the research and then quickly do the job, rather than calling the nurse and then disappear for three hours. I saw my daughter struggling for three more hours because of the research. | | | | | | | |
|  | | | | | | | |
| **Those that didn’t consent** | | | | | | | |
| **Statement** | **Agree n (%)** | | **Neither agree nor disagree n (%)** | | | **Disagree n (%)** | |
| a. The doctor or nurse checked that it was a convenient time to discuss research before discussing FIRST-ABC | 1 | 100% | 0 | 0% | | 0 | 0% |
| b. I was initially surprised to find out that my child had already been entered into FIRST-ABC | 0 | 0% | 1 | 100% | | 0 | 0% |
| c. The information I received about FIRST-ABC was clear and straightforward to understand | 1 | 100% | 0 | 0% | | 0 | 0% |
| d. I understood why consent for my child’s participation in FIRST-ABC was sought after the treatment had been given | 1 | 100% | 0 | 0% | | 0 | 0% |
| e. I had enough opportunity to ask questions about FIRST-ABC | 1 | 100% | 0 | 0% | | 0 | 0% |
| f. I was satisfied with the deferred consent process for FIRST-ABC | missing |  |  |  | |  |  |
| g. It was difficult to take in the information I was given about FIRST-ABC | 0 | 0% | 0 | 0% | | 1 | 100% |
| h. It was difficult to make a decision about FIRST-ABC | 0 | 0% | 0 | 0% | | 1 | 100% |
| i. I made this decision | 1 | 100% | 0 | 0% | | 0 | 0% |
| j. Someone took this decision away from me | 0 | 0% | 0 | 0% | | 1 | 100% |
| k. I was not in control of this decision | 0 | 0% | 0 | 0% | | 1 | 100% |
| l. The decision about the research was inappropriately influenced by others | 0 | 0% | 0 | 0% | | 1 | 100% |

Table S4: Modified COMFORT score and use of sedative agents by treatment group

| **Variables** | **HFNC** | **CPAP** |
| --- | --- | --- |
|  | **N = 59** | **N = 54** |
| **Modified COMFORT score** |  |  |
| Modified COMFORT score completed, n/N (% of all eligible time points) |  |  |
| Group A | 66/117 (56.4) | 49/103 (47.6) |
| Group B |  |  |
| Planned | 75/200 (37.5) | 39/116 (33.6) |
| Rescue | 40/80 (50.0) | 28/120 (23.3) |
| Total | 181/397 (45.6) | 116/339 (34.2) |
|  |  |  |
| Patients with at least one modified COMFORT score completed, n/N (%) |  |  |
| Group A | 10/15 (66.7) | 7/12 (58.3) |
| Group B |  |  |
| Planned | 19/27 (70.4) | 10/17 (58.8) |
| Rescue | 7/11 (63.6) | 6/17 (35.3) |
| Total | 36/53 (67.9) | 23/46 (50.0) |
|  | | |
| Modified COMFORT score (Hour 1-6) by tolerating randomised treatment at individual time points, mean (SD) [N] | | |
| Group A |  |  |
| Yes | 16.6 (4.2) [47] | 15.0 (2.9) [31] |
| No | N=1 | N=0 |
| Group B |  |  |
| Planned |  |  |
| Yes | 16.4 (3.5) [44] | 14.9 (2.8) [25] |
| No | N=0 | N=0 |
| Rescue |  |  |
| Yes | 15.8 (3.8) [23] | 14.0 (2.6) [12] |
| No | 16.7 (1.5) [3] | 19.0 (4.4) [3] |
| Total |  |  |
| Yes | 16.4 (3.8) [114] | 14.8 (2.8) [68] |
| No | 15.3 (3.1) [4] | 19.0 (4.4) [3] |
|  |  |  |
| Use of sedative agents, n/N (%) |  |  |
| Group A | 6/15 (40.0) | 3/12 (25.0) |
| Group B |  |  |
| Planned | 7/27 (25.9) | 6/18 (33.3) |
| Rescue | 7/12 (58.3) | 9/18 (50.0) |
| Total | 20/54 (37.0) | 18/48 (37.5) |

Table S5: Adverse events by treatment group

| **Variables** | **HFNC** | **CPAP** |
| --- | --- | --- |
|  | **N = 59** | **N = 54** |
| **Adverse events^a^** |  |  |
| Pneumothorax, n/N (%) |  |  |
| Group A | 0/16 (0.0) | 0/13 (0.0) |
| Group B |  |  |
| Planned | 0/30 (0.0) | 0/22 (0.0) |
| Rescue | 0/13 (0.0) | 0/19 (0.0) |
| Total | 0/59 (0.0) | 0/54 (0.0) |
|  |  |  |
| Pneumomediastinum, n/N (%) |  |  |
| Group A | 0/16 (0.0) | 0/13 (0.0) |
| Group B |  |  |
| Planned | 0/30 (0.0) | 0/22 (0.0) |
| Rescue | 0/13 (0.0) | 0/19 (0.0) |
| Total | 0/59 (0.0) | 0/54 (0.0) |
|  |  |  |
| Subcutaneous emphysema, n/N (%) |  |  |
| Group A | 0/16 (0.0) | 0/13 (0.0) |
| Group B |  |  |
| Planned | 0/30 (0.0) | 0/22 (0.0) |
| Rescue | 0/13 (0.0) | 0/19 (0.0) |
| Total | 0/59 (0.0) | 0/54 (0.0) |
|  |  |  |
| Abdominal distension, n/N (%) |  |  |
| Group A | 1/16 (6.3) | 0/13 (0.0) |
| Group B |  |  |
| Planned | 0/30 (0.0) | 0/22 (0.0) |
| Rescue | 0/13 (0.0) | 1/19 (5.3) |
| Total | 1/59 (1.7) | 1/54 (1.9) |
|  |  |  |
| Nasal trauma, n/N (%) |  |  |
| Group A | 0/16 (0.0) | 0/13 (0.0) |
| Group B |  |  |
| Planned | 0/30 (0.0) | 1/22 (4.5) |
| Rescue | 0/13 (0.0) | 1/19 (5.3) |
| Total | 0/59 (0.0) | 2/54 (3.7) |
|  |  |  |
| Facial trauma, n/N (%) |  |  |
| Group A | 0/16 (0.0) | 0/13 (0.0) |
| Group B |  |  |
| Planned | 1/30 (3.3) | 0/22 (0.0) |
| Rescue | 0/13 (0.0) | 0/19 (0.0) |
| Total | 1/59 (1.7) | 0/54 (0.0) |
|  |  |  |
| Facial thermal injury, n/N (%) |  |  |
| Group A | 1/16 (6.3) | 0/13 (0.0) |
| Group B |  |  |
| Planned | 0/30 (0.0) | 0/22 (0.0) |
| Rescue | 0/13 (0.0) | 0/19 (0.0) |
| Total | 1/59 (1.7) | 0/54 (0.0) |
|  |  |  |
| Respiratory/Cardiac arrest, n/N (%) |  |  |
| Group A | 0/16 (0.0) | 0/13 (0.0) |
| Group B |  |  |
| Planned | 0/30 (0.0) | 2/22 (9.1) |
| Rescue | 0/13 (0.0) | 1/19 (5.3) |
| Total | 0/59 (0.0) | 3/54 (5.6) |
|  |  |  |
| Aspiration, n/N (%) |  |  |
| Group A | 0/16 (0.0) | 1/13 (7.7) |
| Group B |  |  |
| Planned | 0/30 (0.0) | 0/22 (0.0) |
| Rescue | 0/13 (0.0) | 0/19 (0.0) |
| Total | 0/59 (0.0) | 1/54 (1.9) |
|  |  |  |
| Other^b^, n (%) |  |  |
| Group A | 1/16 (6.3) | 2/13 (15.4) |
| Group B |  |  |
| Planned | 3/30 (10.0) | 0/22 (0.0) |
| Rescue | 1/13 (7.7) | 0/19 (0.0) |
| Total | 5/59 (8.5) | 2/54 (3.7) |

n: Number of patients; %: Percentage of patients; N: Total number of patients

^a^4 adverse events were reported to be severe: 3 respiratory/cardiac arrest (CPAP, Group B); 1 pulmonary haemorrhage (HFNC, Group B)

^b^Other reported adverse events were: 3 extravasion injury (2 HFNC, Group B; 1 CPAP, Group A); 1 haematuria & pressure sore (HFNC, Group A); 1 nappy rash (CPAP, Group A); 1 pericardial effusion (HFNC, Group B); 1 pulmonary haemorrhage (HFNC, Group B)

Table S6: Reasons and timing for crossover and escalation to intubation and invasive ventilation within 72 hours by treatment group

| **Reasons** | **HFNC** | **CPAP** |
| --- | --- | --- |
|  | **N = 59** | **N = 54** |
| **Crossover, n/N (%)** |  |  |
| Failure to improve hypoxia | 2 (3.4) | 0 |
| Failure to improve respiratory acidosis | 1 (1.7) | 0 |
| Failure to improve respiratory distress | 12 (20.3) | 0 |
| Intolerance to randomised treatment | 0 | 6 (11.1) |
| Other | 1 (1.7) | 2 (3.7) |
| Total | 16 (27.1) | 8 (14.8) |
|  |  |  |
| Median time from randomisation to crossover, hours (IQR) | 5.5 (3.1, 21.2) | 2.4 (0.8, 18.9) |
|  |  |  |
| **Intubation and invasive ventilation, n/N (%)** |  |  |
| Failure to improve hypoxia | 3 (5.1) | 2 (3.7) |
| Failure to improve respiratory acidosis | 1 (1.7) | 1 (1.9) |
| Failure to improve respiratory distress | 8 (13.6) | 6 (11.1) |
| Intolerance to randomised treatment | 0 | 0 |
| Other | 2 (3.4) | 1 (1.9) |
| Unknown | 1 (1.7) | 0 |
| Total | 15 (25.4) | 10 (18.5) |
|  |  |  |
| Median time from randomisation to intubation, hours (IQR) | 5.5 (1.7, 17.3) | 3.3 (1.6, 19.8) |
